# Online adaptive magnetic resonance guided radiotherapy for pancreatic cancer: state of the art, pearls and pitfalls

**DOI:** 10.1186/s13014-019-1275-3

**Published:** 2019-04-29

**Authors:** Luca Boldrini, Davide Cusumano, Francesco Cellini, Luigi Azario, Gian Carlo Mattiucci, Vincenzo Valentini

**Affiliations:** 1 Dipartimento di Diagnostica per immagini, Radioterapia Oncologica ed Ematologia, UOC Radioterapia Oncologica, Fondazione Policlinico Universitario “A. Gemelli” IRCCS, Roma, Italia; 2 Dipartimento di Diagnostica per immagini, Radioterapia Oncologica ed Ematologia, UOC Fisica Sanitaria, Fondazione Policlinico Policlinico Universitario “A. Gemelli” IRCCS, Roma, Italia

**Keywords:** Online adaptive radiotherapy, MR-guided radiotherapy, Pancreatic cancer

## Abstract

**Background:**

Different studies have proved in recent years that hypofractionated radiotherapy (RT) improves overall survival of patients affected by locally advanced, unresectable, pancreatic cancer.

The clinical management of these patients generally leads to poor results and is considered very challenging, due to different factors, heavily influencing treatment delivery and its outcomes.

Firstly, the dose prescribed to the target is limited by the toxicity that the highly radio-sensitive organs at risk (OARs) surrounding the disease can develop. Treatment delivery is also complicated by the significant inter-fractional and intra-fractional variability of therapy volumes, mainly related to the presence of hollow organs and to the breathing cycle.

**Main body of the abstract:**

The recent introduction of magnetic resonance guided radiotherapy (MRgRT) systems leads to the opportunity to control most of the aforementioned sources of uncertainty influencing RT treatment workflow in pancreatic cancer.

MRgRT offers the possibility to accurately identify radiotherapy volumes, thanks to the high soft-tissue contrast provided by the Magnetic Resonance imaging (MRI), and to monitor the tumour and OARs positions during the treatment fraction using a high-temporal cine MRI.

However, the main advantage offered by the MRgRT is the possibility to online adapt the RT treatment plan, changing the dose distribution while the patient is still on couch and successfully addressing most of the sources of variability.

**Short conclusion:**

Aim of this study is to present and discuss the state of the art, the main pitfalls and the innovative opportunities offered by online adaptive MRgRT in pancreatic cancer treatment.

## Background

Pancreatic cancer represents one of the most aggressive tumours with a 5 years overall survival (OS) rate ranging from 5 to 20%. Surgery still represents the most valuable therapeutic option, although only 20% of patients appears to be candidate for resection at the time of diagnosis [1, 2].

The large majority of patients affected by pancreatic cancer presents with locally advanced unresectable tumours, whose clinical management is complex and characterized by very poor prognosis [3].

Conflicting results regarding the benefit of chemotherapy, radiotherapy (RT) and their combination (CRT) in pancreatic cancer have been reported in literature: the LAP07 randomized clinical trial observed no significant difference in overall survival with CRT compared with chemotherapy alone, while the GERCOR study suggested that sequential CRT could improve survival of pancreatic cancer patients compared with chemotherapy alone; also the Eastern Cooperative Oncology Group (ECOG) trial demonstrated the superiority of the gemcitabine plus radiotherapy arm compared to gemcitabine alone, even if severe toxicity rate was higher [4–6].

Furthermore, different studies have demonstrated that hypofractionated RT combined or not with chemotherapy and administered with different timing may improve OS, even if the risk of toxicity for the surrounding organs at risk (OARs) still remains a strong dose limiting factor in this setting [7–12].

Treatment management is also affected by the difficulty to accurately identify RT volumes due to the poor soft-tissue contrast offered in the abdominal site by the ionising radiations based imaging techniques generally used in RT standard delivery technologies, such as computed tomography (CT) and the Cone Beam Computed Tomography (CBCT).

Motion management represents another crucial issue to achieve a safe and efficient delivery of the treatment, especially considering how physiological movements (e.g. breathing cycle) can dislocate both target volumes and OARs during treatment delivery. Karava et al. have recently estimated respiratory-induced pancreatic motion in 12 patients using 4D-CT: mean displacement of 2 mm in antero-posterior (AP), 4.8 mm in inferior-superior (IS) and 1.3 mm in left-right (LR) direction were reported, values that can be hardly managed by the usual target margins. Other authors reported pancreatic movements up to 23 mm in IS, 11 mm in AP and 7 mm in LR directions [13–15].

This significant displacement of therapy volumes may be linked to both an inter-fractional component related to the anatomical variability of the surrounding OARs (above all, hollow organs like stomach or duodenum and the highly movable bowel loops), and an intra-fractional one, with abdominal anatomy being affected by breathing cycle phases and physiological movements throughout the delivery of RT fraction.

One of the most promising delivery techniques is represented by stereotactic body radiotherapy (SBRT), considered either as an exclusive approach or in combination with other therapeutic approaches.

In this context, the new RT hybrid systems that join radiation delivery units (both Cobalt sources and Linac) with Magnetic Resonance Imaging (MRI) scanners, offer various significant advantages for RT treatment delivery, especially for the treatment of upper gastro-intestinal malignancies and, particularly, for pancreatic cancer.

Unity (Elekta, Stockholm, Sweden) uses a 1.5 T MRI scanner with a 7 MV Flattening Filter Free (FFF) Linac, while MRIdian (ViewRay, Cleveland, Ohio) joins 0.35 T MRI scanner with three ^60^Co γ-ray sources or a 6 MV FFF Linac for radiation delivery [16–18].

As for the irradiation technique, none of the current devices supports highly conformal solutions, such as volumetric modulated arc radiotherapy (VMAT) or sliding windows intensity modulated radiotherapy (IMRT) and treatments are delivered with a step-and-shoot IMRT approach.

The most significant advantage offered by the innovative MR guided radiotherapy (MRgRT) approach is represented by the superior soft-tissue contrast offered by MRI, that allows a more precise identification of the therapy volumes respect to the one reachable using CT images, and the subsequent reduction of the clinical target volume (CTV) to planning target volume (PTV) margin expansion.

This reduction leads to remarkable dosimetric advantages in terms of dose reduction to the OARs, maintaining optimal dose coverage to the target, as demonstrated in recent planning studies [19, 20].

The MR images provided by these hybrid machines can be used in three main clinical applications, covering the whole RT treatment workflow.

### Positioning and alignment imaging

The higher morphological quality of MR images improves the visualization and the delineation of therapy volumes if compared to standard positioning imaging [21].

### Real time cine imaging for gating purposes throughout the treatment

Treatment gating protocols can be directly applied to target volumes, surrogate target volumes (especially if target is not clearly visible on positioning image) or even to OARs in order to optimize their sparing [22, 23] .

### Advanced online adaptive applications

The hybrid units allow to perform advanced online adaptive applications in which the therapy volumes are re-contoured every day with the patient being on the couch, the dose distribution is quickly adapted taking into account the occurred anatomical variations and an optimized plan is then delivered according to the most convenient configuration.

This strategy, defined as online magnetic resonance-guided adaptive radiotherapy (MRgART), allows to safely deliver high doses to the target, minimising the dose to the OARs and successfully managing organ motion [24].

### MRgART for pancreatic cancer: clinics

Numerous authors have confirmed the feasibility and safety of SBRT in pancreatic cancer with standard linacs, achieving 1-year local control of 80% in locally advanced pancreatic cancer (LAPC), even if the first experiences were burdened by high rates of ≥3 grade gastrointestinal toxicity, representing a significant dose limiting factor [25–30].

The introduction of IMRT, advanced motion management solutions (i.e. respiratory gating) and Image Guided Radiotherapy (IGRT) techniques (i.e. CBCT, CT-on-rails) contributed in reducing gastrointestinal side effects and escalating the dose to the target volumes, reaching higher biological equivalent doses [25, 31].

Even if the first dosimetric studies and clinical results are promising, the use of protons and other particles for LAPC treatment is to be explored and photon therapy still represents the standard of care [32, 33].

In this context, owing to its technological and advanced imaging characteristics, MRgART can offer significant advantages in the clinical management of pancreatic cancer patients [34].

The first cohort of patients treated with MRgART was described by Henke et al: 20 oligometastatic (three or less lesions) or unresectable patients: ten of which suffered from primary or secondary liver lesions, five from pancreatic cancer (3 recurrences and 2 primary) and five from abdominal secondary nodal lesions [35].

The prescribed dose was 50 Gy, delivered in five fractions, for all plans and primary endpoint of the study was to deliver adaptive treatment in less than 80 min per fraction for > 75% of cases.

Hard constraints were applied to reduce toxicity (see proper section in Table 1) and 75% of the fractions was adapted to reverse violations (mainly for small bowel) and prospectively reduce gastrointestinal toxicity.

**Table 1:** Organs at risk dose constraints for Stereotactic Body Radiation Therapy (SBRT) in LAPC patients as proposed by Bohoudi et al. [36] and Henke et al. [35]

OARs	Bohoudi et al [36]	Henke et al [35]
Liver	V12Gy < 50%	700 cc < 20Gy (uninvolved liver) V25Gy < 33% Mean < 20Gy
Duodenum	V33Gy ≤ 1 cc V25Gy < 20 cc	V35Gy ≤ 0.5 cc
Stomach	V33Gy ≤ 1 cc V25Gy < 20 cc	V33Gy ≤ 0.5 cc
Bowel Bag	V33Gy ≤ 1 cc V25Gy < 20 cc	V30Gy ≤ 0.5 cc (Small Bowel) V35Gy ≤ 0.5 cc (Large Bowel)
Cord	N/A	V25Gy < 0.5 cc
Kidneys (combined)	V12Gy < 25%	Mean Dose <18Gy
Heart/Pericardium	N/A	V32Gy < 15 cc

*OARs* organs at risk, *Gy* Gray, *cc* cubic centimetres

Plan adaptation defined an improvement of PTV coverage in 57% of the cases, while dose reduction was needed to respect OARs constraints in the remaining cases.

Dose escalation beyond the originally prescribed dose was achieved only in three liver patients but was never observed for the other abdominal sites. One-year OS rate was 75%: two out of the three patients with recurrent LAPC showed progression of the disease according to RECIST criteria, with a median follow up of 15 months (7.5–21 months).

The two patients with primary pancreatic lesions were both alive with no progression after 14 months of follow up.

The results in terms of toxicity and quality of life (QoL) were also encouraging: no cases of ≥ G3 toxicities (CTCAE v.4), one case of G2 ulcer outside the irradiation field and no significant modification of QoL parameters were observed during therapy and after a median follow up of 15 months [35].

This experience suggests that MRgART may be feasible for upper gastrointestinal malignancies (both for primary disease presentation or in oligometastatic setting) and that pancreatic cancer can represent a good candidate for this innovative approach.

### MRgART for pancreatic cancer: physics

The clinical evidence to date available for pancreatic MRgART applications is based on the use of hybrid machines equipped with low Tesla on-board MR scanners [17, 35, 36].

**Fig. 1:**
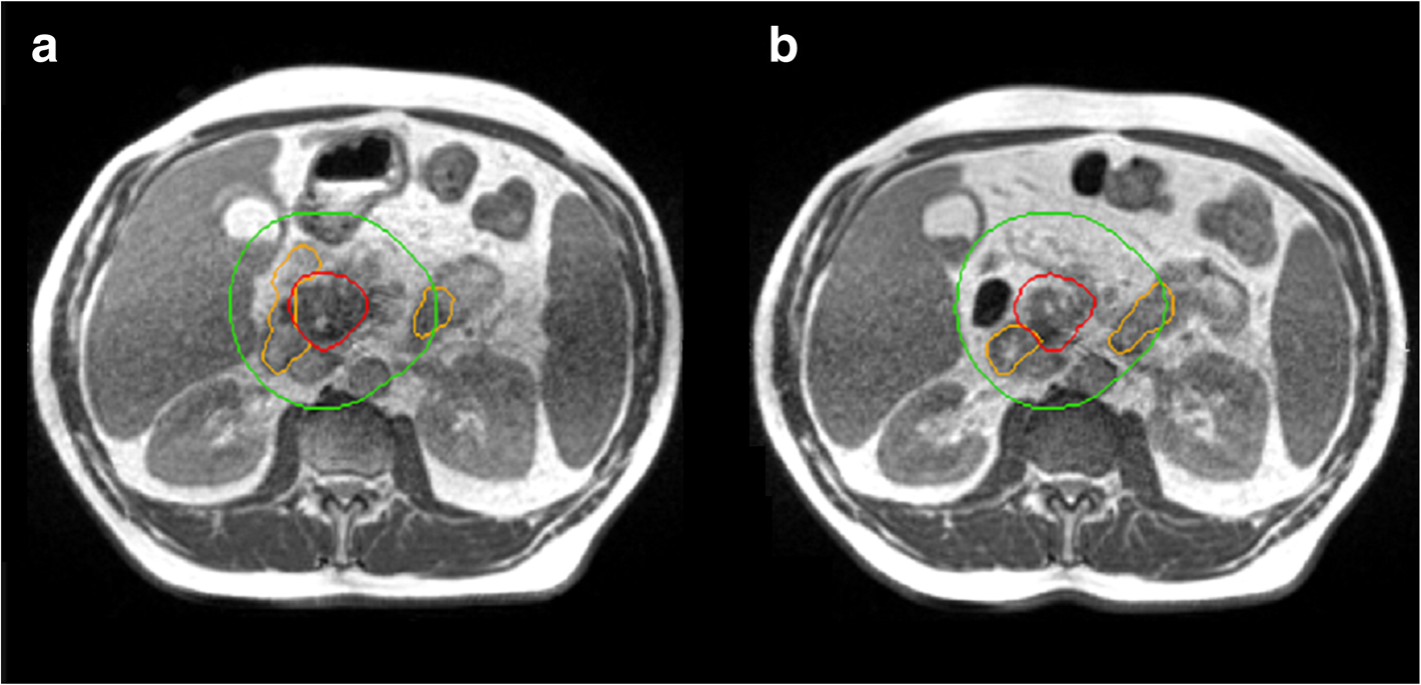
Example of inter-fraction variability for the case of upper abdomen, as occurred between two consecutive days of treatment in the same patient’s preparation conditions. The duodenum position (orange) significantly changes its position respect to the pancreatic cancer (red). A 3 cm wide region surrounding the GTV is reported in green

Thanks to the enhanced soft tissue contrast, low Tesla MR images represent an excellent support for therapy volumes segmentation (see Fig. 1), especially in those sites where it is hard to precisely identify targets and OAR in standard CT based image guided radiotherapy, due to soft tissue isodensity (i.e. CBCT) [37] .

**Fig. 2:**
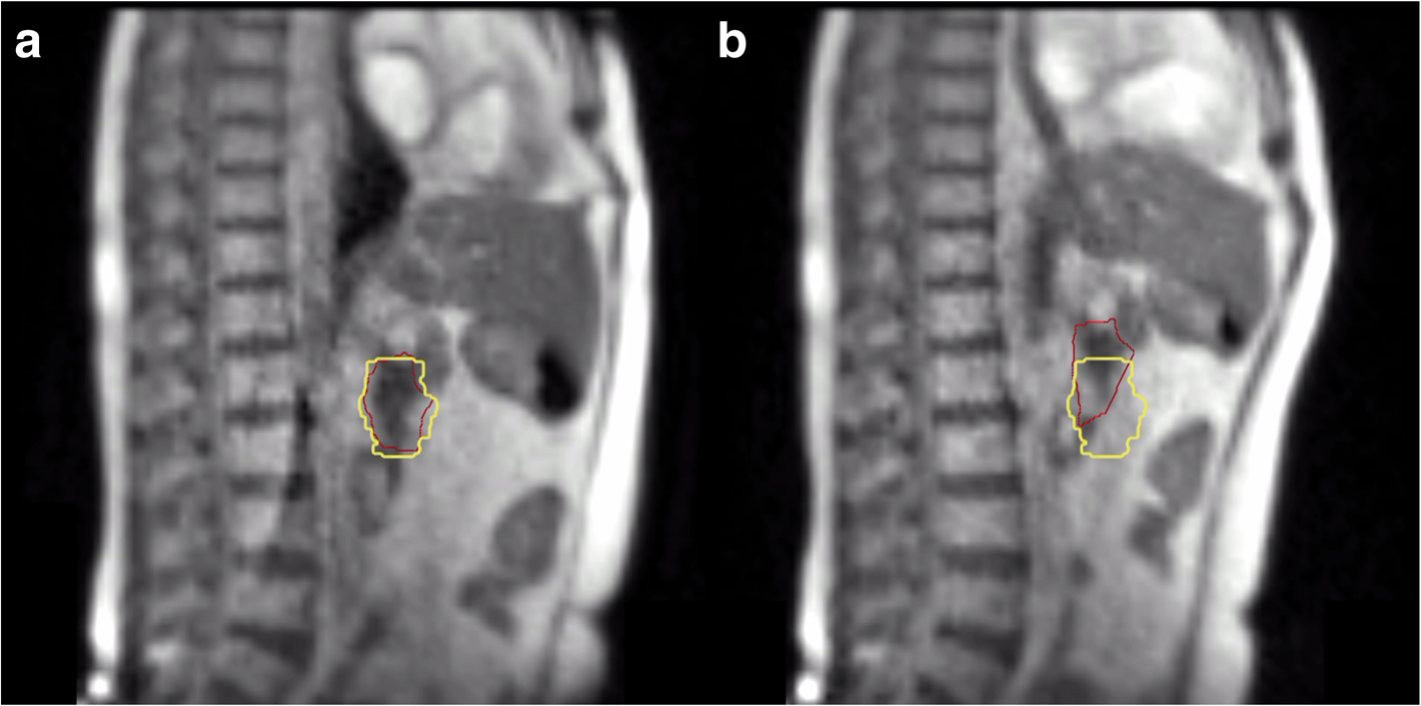
Intra-fraction motion management by means of cine MR. The treatment is delivered only when the target structure (in red) is inside the defined boundary region (in yellow), as described in part (**a**). In the case of part (**b**), treatment delivery stops until the right volume position is reached

In addition to the advantages relative to segmentation and positioning imaging quality, the possibility of monitoring treatment delivery using a real-time cine MRI represents another favourable opportunity offered by the MRgRT systems (see Fig. 2).

The use of gating cine MRI allows a direct visualisation of therapy volumes (both targets and OARs) with a temporal frequency of 4 images per second. Its superiority respect to the use of implanted markers or other external surrogates has been demonstrated in different experiences in the case of SBRT treatments [38, 39].

Furthermore, the use of low tesla MRI ensures higher contrast to noise ratio (CNR) between the target (i.e.: tumour) and its background, respect to the one obtainable using a high magnetic field in real time tumour tracking, and successfully reduces the artefacts influencing MRI’s spatial integrity [40].

Nevertheless, the timespan for a fully online adaptive workflow (i.e. evaluation of initial plan, contouring, re-evaluation of the initial plan on the new contours, re-optimization) still represents a limiting factor in the adaptive workflow: Lamb et al have estimated a median execution fraction time of 54 min on 80 cases, with contouring being the most time spending step (mean time: 22 min), against only a couple of minutes of Monte Carlo based dose calculation [41]. On the other hand, it is notable that in their first experience Henke et al. observed that the adapted treatment was overall well tolerated, despite a mean duration of 80 min per fraction [35].

Two different optimisation approaches have been recently proposed in order to speed up and standardise the online MRgART workflow in LAPC.

Olberg et al suggest to group all the OARs surrounding the GTV in a single structure and then crop the PTV by 3 mm to this volume, while Bohoudi et al propose the “stereotactic MR-guided adaptive radiotherapy” (SMART) approach, consisting in the combination of all the surrounding OARs in different optimisation regions located at 1, 2 and 3 cm from the PTV edge [36, 42].

In both studies 40 Gy in 5 fractions were delivered to the PTV, obtained applying an isotropic 3 mm expansion from the GTV. The constraints applied in the SMART approach to the OARs are reported in the right column of Table 1 [36].

The strategies to date adopted for patient specific QA of the re-optimized plans consist in a secondary dose calculation based on an independent algorithm, even if alternative approaches (e.g. MR compatible Electron Portal Imaging Detector (EPID)-based QA workflows) are currently under investigation [43].

Besides the need of a robust dose QA process standardization, another potential pitfall of the MRgART workflow is represented by dose summation solutions that should be able to sum the doses actually delivered in the single treatment fractions taking into account the daily change of both anatomy and dose distribution. Although several strategies have been proposed to this end, mainly based on the application of deformable image registration algorithms, the definition of a clear and common strategy is still far to be individuated [44].

## Conclusions

### Open issues and future perspectives

The expected developments of MRgRT in terms of delivery technology improvements (i.e. collimator leaves width progressive reduction; more accurate optimization software; volumetric dynamic delivery possibilities; new gating algorithms) may overcome the current pitfalls of MRgRT and open new perspectives for the clinical management of LAPC patients.

More robust dose accumulation algorithms may allow MRgRT treatment’s safety and reliability improvements, while further progresses in calculation and the application of artificial intelligence based autosegmentation models will significantly reduce the fully online MRgART workflow timespan, enhancing contours quality, reducing “prior to delivery” anatomical uncertainties and making treatments better tolerated, facilitating patient’s compliance and endurance in treatment position [44].

Furthermore, functional imaging applications (e.g. Diffusion Weighted Imaging) on MR positioning and delivery imaging could open new scenarios in RT target definition and therapy volume biological characterization throughout the treatment (i.e. early response or toxicity onset assessment), while its radiomics and quantitative analyses may allow an innovative, fully personalized, therapeutic approach [45, 46].

In conclusion, promising results in terms of tumour control, toxicity occurrence and survival rates have been reported in literature for pancreatic cancer MRgRT, even if based on small patients samples due to the scarcity of active hybrid units. These results may be further improved thanks to the ongoing research protocols exploring dose escalation and toxicity characterization and to the awareness reached through a more experienced use of the available imaging tools and gating solutions [47].

## Abbreviations

AP: Antero-posterior
B: Magnetic field
CBCT: Cone Beam Computed Tomography
CRT: Chemoradiotherapy
CT: Computed tomography
CTV: Clinical Target Volume
ECOG: Eastern Cooperative Oncology Group
ED: Electron Density
EPID: Electron Portal Imaging Detectors
FFF: Flattening Filter Free
GPU: Graphical Power Unit
GTV: Gross Tumour Volume
IGRT: Image guided radiotherapy
IS: Inferior-superior
LAPC: Locally advanced pancreatic cancer
LR: Left-right
MLC: Multi Leaf Collimator
MR: Magnetic Resonance
MRgART: Magnetic Resonance-guided adaptive radiotherapy
MRgRT: Magnetic Resonance-guided radiotherapy
MRI: Magnetic Resonance Imaging
OARs: Organs at risk
OS: Overall survival
PTV: Planning Target Volume
QA: Quality Assurance
QoL: Quality of Life
RT: Radiotherapy
SBRT: Stereotactic Body Radiation Therapy
SMART: Stereotactic MR-guided adaptive radiotherapy
TPS: Treatment planning system
VMAT: Volumetric modulated arc radiotherapy

## References

[1] Herman JM, Wild AT, Wang H, Tran PT, Chang KJ, Taylor GE (2013). Randomized phase III multi-institutional study of TNFerade biologic with fluorouracil and radiotherapy for locally advanced pancreatic cancer: final results. J Clin Oncol Off J Am Soc Clin Oncol.

[2] Malvezzi M, Bertuccio P, Rosso T, Rota M, Levi F, La Vecchia C (2015). European cancer mortality predictions for the year 2015: does lung cancer have the highest death rate in EU women?. Ann Oncol.

[3] Hidalgo M (2010). Pancreatic cancer. N Engl J Med.

[4] Hammel P, Huguet F, van Laethem J-L, Goldstein D, Glimelius B, Artru P (2016). Effect of Chemoradiotherapy vs chemotherapy on survival in patients with locally advanced pancreatic Cancer controlled after 4 months of gemcitabine with or without Erlotinib: the LAP07 randomized clinical trial. JAMA.

[5] Huguet F, André T, Hammel P, Artru P, Balosso J, Selle F (2007). Impact of chemoradiotherapy after disease control with chemotherapy in locally advanced pancreatic adenocarcinoma in GERCOR phase II and III studies. J Clin Oncol Off J Am Soc Clin Oncol.

[6] Loehrer PJ, Feng Y, Cardenes H, Wagner L, Brell JM, Cella D (2011). Gemcitabine alone versus gemcitabine plus radiotherapy in patients with locally advanced pancreatic cancer: an eastern cooperative oncology group trial. J Clin Oncol Off J Am Soc Clin Oncol.

[7] Ben-Josef E, Schipper M, Francis IR, Hadley S, Ten-Haken R, Lawrence T (2012). A phase I/II trial of intensity modulated radiation (IMRT) dose escalation with concurrent fixed-dose rate gemcitabine (FDR-G) in patients with unresectable pancreatic cancer. Int J Radiat Oncol Biol Phys.

[8] Lominska CE, Unger K, Nasr NM, Haddad N, Gagnon G (2012). Stereotactic body radiation therapy for reirradiation of localized adenocarcinoma of the pancreas. Radiat Oncol.

[9] De Bari B, Porta L, Mazzola R, Alongi F, Wagner AD, Schäfer M (2016). Hypofractionated radiotherapy in pancreatic cancer: lessons from the past in the era of stereotactic body radiation therapy. Crit Rev Oncol Hematol.

[10] Reese AS, Lu W, Regine WF (2014). Utilization of intensity-modulated radiation therapy and image-guided radiation therapy in pancreatic cancer: is it beneficial?. Semin Radiat Oncol.

[11] Bockbrader M, Kim E (2009). Role of intensity-modulated radiation therapy in gastrointestinal cancer. Expert Rev Anticancer Ther.

[12] Didolkar MS, Coleman CW, Brenner MJ, Chu KU, Olexa N, Stanwyck E (2010). Image-guided stereotactic radiosurgery for locally advanced pancreatic adenocarcinoma results of first 85 patients. J Gastrointest Surg.

[13] Karava K, Ehrbar S, Riesterer O, Roesch J, Glatz S, Klöck S (2017). Potential dosimetric benefits of adaptive tumor tracking over the internal target volume concept for stereotactic body radiation therapy of pancreatic cancer. Radiat Oncol.

[14] Heerkens HD, van Vulpen M, van den Berg CAT, Tijssen RHN, Crijns SPM, Molenaar IQ (2014). MRI-based tumor motion characterization and gating schemes for radiation therapy of pancreatic cancer. Radiother Oncol.

[15] Knybel L, Cvek J, Otahal B, Jonszta T, Molenda L, Czerny D (2014). The analysis of respiration-induced pancreatic tumor motion based on reference measurement. Radiat Oncol.

[16] Lagendijk JJW, Raaymakers BW, van Vulpen M (2014). The magnetic resonance imaging-linac system. Semin Radiat Oncol.

[17] Mutic S, Dempsey JF (2014). The ViewRay system: magnetic resonance-guided and controlled radiotherapy. Semin Radiat Oncol.

[18] Raaymakers BW, Lagendijk JJW, Overweg J, Kok JGM, Raaijmakers AJE, Kerkhof EM (2009). Integrating a 1.5 T MRI scanner with a 6 MV accelerator: proof of concept. Phys Med Biol.

[19] Park JM, Park S-Y, Kim J-I, Kang H-C, Choi CH (2017). A comparison of treatment plan quality between tri-co-60 intensity modulated radiation therapy and volumetric modulated arc therapy for cervical cancer. Phys Med.

[20] Boldrini L, Placidi E, Dinapoli N, Azario L, Cellini F, Massaccesi M (2018). Hybrid tri-co-60 MRI radiotherapy for locally advanced rectal cancer: an in silico evaluation. Tech Innov Patient Support Radiat Oncol.

[21] Kupelian P, Sonke J-J (2014). Magnetic resonance-guided adaptive radiotherapy: a solution to the future. Semin Radiat Oncol.

[22] Boldrini L, Cellini F, Manfrida S, Chiloiro G, Teodoli S, Cusumano D (2018). Use of indirect target gating in magnetic resonance-guided liver stereotactic body radiotherapy: case report of an Oligometastatic patient. Cureus.

[23] Massaccesi M, Cusumano D, Boldrini L, Dinapoli N, Fionda B, Teodoli S, et al. A new frontier of image guidance. Organs at risk avoidance with MRI-guided respiratory-gated intensity modulated radiotherapy; technical note and report of a case. J Appl Clin Med Phys. in press. 10.1002/acm2.12575 PMC6560311 31055870

[24] Wu QJ, Li T, Wu Q, Yin F-F (2011). Adaptive radiation therapy: technical components and clinical applications. Cancer J.

[25] Ng Sweet Ping, Koay Eugene J. (2018). Current and emerging radiotherapy strategies for pancreatic adenocarcinoma: stereotactic, intensity modulated and particle radiotherapy. Annals of Pancreatic Cancer.

[26] Schellenberg D, Goodman KA, Lee F, Chang S, Kuo T, Ford JM (2008). Gemcitabine chemotherapy and single-fraction stereotactic body radiotherapy for locally advanced pancreatic cancer. Int J Radiat Oncol Biol Phys.

[27] Chang DT, Schellenberg D, Shen J, Kim J, Goodman KA, Fisher GA (2009). Stereotactic radiotherapy for unresectable adenocarcinoma of the pancreas. Cancer.

[28] Chuong MD, Springett GM, Freilich JM, Park CK, Weber JM, Mellon EA (2013). Stereotactic body radiation therapy for locally advanced and borderline resectable pancreatic cancer is effective and well tolerated. Int J Radiat Oncol Biol Phys.

[29] Gurka MK, Kim C, He AR, Charabaty A, Haddad N, Turocy J (2017). Stereotactic body radiation therapy (SBRT) combined with chemotherapy for Unresected pancreatic adenocarcinoma. Am J Clin Oncol.

[30] Pollom EL, Alagappan M, von Eyben R, Kunz PL, Fisher GA, Ford JA (2014). Single- versus multifraction stereotactic body radiation therapy for pancreatic adenocarcinoma: outcomes and toxicity. Int J Radiat Oncol Biol Phys.

[31] Bittner M-I, Grosu A-L, Brunner TB (2015). Comparison of toxicity after IMRT and 3D-conformal radiotherapy for patients with pancreatic cancer - a systematic review. Radiother Oncol.

[32] Thompson RF, Mayekar SU, Zhai H, Both S, Apisarnthanarax S, Metz JM (2014). A dosimetric comparison of proton and photon therapy in unresectable cancers of the head of pancreas. Med Phys.

[33] Shinoto M, Ebner DK, Yamada S (2016). Particle radiation therapy for gastrointestinal cancers. Curr Oncol Rep.

[34] El-Bared Nancy, Portelance Lorraine, Spieler Benjamin O., Kwon Deukwoo, Padgett Kyle R., Brown Karen M., Mellon Eric A. (2019). Dosimetric Benefits and Practical Pitfalls of Daily Online Adaptive MRI-Guided Stereotactic Radiation Therapy for Pancreatic Cancer. Practical Radiation Oncology.

[35] Henke L, Kashani R, Robinson C, Curcuru A, DeWees T, Bradley J (2018). Phase I trial of stereotactic MR-guided online adaptive radiation therapy (SMART) for the treatment of oligometastatic or unresectable primary malignancies of the abdomen. Radiother Oncol.

[36] Bohoudi O, Bruynzeel AME, Senan S, Cuijpers JP, Slotman BJ, Lagerwaard FJ (2017). Fast and robust online adaptive planning in stereotactic MR-guided adaptive radiation therapy (SMART) for pancreatic cancer. Radiother Oncol.

[37] Noel CE, Parikh PJ, Spencer CR, Green OL, Hu Y, Mutic S (2015). Comparison of onboard low-field magnetic resonance imaging versus onboard computed tomography for anatomy visualization in radiotherapy. Acta Oncol.

[38] Brandner ED, Chetty IJ, Giaddui TG, Xiao Y, Huq MS (2017). Motion management strategies and technical issues associated with stereotactic body radiotherapy of thoracic and upper abdominal tumors: a review from NRG oncology. Med Phys.

[39] Dieterich S, Green O, Booth J (2018). SBRT targets that move with respiration. Phys Med.

[40] Wachowicz K, De Zanche N, Yip E, Volotovskyy V, Fallone BG (2016). CNR considerations for rapid real-time MRI tumor tracking in radiotherapy hybrid devices: effects of B0 field strength. Med Phys.

[41] Lamb J, Cao M, Kishan A, Agazaryan N, Thomas DH, Shaverdian N (2017). Online adaptive radiation therapy: implementation of a new process of care. Cureus.

[42] Olberg S, Green O, Cai B, Yang D, Rodriguez V, Zhang H (2018). Optimization of treatment planning workflow and tumor coverage during daily adaptive magnetic resonance image guided radiation therapy (MR-IGRT) of pancreatic cancer. Radiat Oncol.

[43] Li HH, Rodriguez VL, Green OL, Hu Y, Kashani R, Wooten HO (2015). Patient-specific quality assurance for the delivery of (60)co intensity modulated radiation therapy subject to a 0.35-T lateral magnetic field. Int J Radiat Oncol Biol Phys.

[44] Simon A, Nassef M, Rigaud B, Cazoulat G, Castelli J, Lafond C (2015). Roles of deformable image registration in adaptive RT: from contour propagation to dose monitoring. Conf Proc IEEE Eng Med Biol Soc.

[45] Shaverdian N, Yang Y, Hu P, Hart S, Sheng K, Lamb J (2017). Feasibility evaluation of diffusion-weighted imaging using an integrated MRI-radiotherapy system for response assessment to neoadjuvant therapy in rectal cancer. Br J Radiol.

[46] Boldrini Luca, Cusumano Davide, Chiloiro Giuditta, Casà Calogero, Masciocchi Carlotta, Lenkowicz Jacopo, Cellini Francesco, Dinapoli Nicola, Azario Luigi, Teodoli Stefania, Gambacorta Maria Antonietta, De Spirito Marco, Valentini Vincenzo (2018). Delta radiomics for rectal cancer response prediction with hybrid 0.35 T magnetic resonance-guided radiotherapy (MRgRT): a hypothesis-generating study for an innovative personalized medicine approach. La radiologia medica.

[47] Daily Online Adaptation Versus localization for MRI-guided SBRT for Unresectable primary or Oligometastatic abdominal malignancies - full text view - ClinicalTrials.gov [Internet]. [cited 2018 Jul 24]. Available from: https://clinicaltrials.gov/ct2/show/NCT02950025

